# Current Operative Management of Breast Cancer: An Age of Smaller Resections and Bigger Cures

**DOI:** 10.1155/2012/516417

**Published:** 2011-12-18

**Authors:** Jack W. Rostas, Donna Lynn Dyess

**Affiliations:** Department of Surgery, Mitchell Cancer Institute, University of South Alabama, 1660 Springhill Avenue Mobile, AL 36604, USA

## Abstract

Surgical resection was the first effective treatment for breast cancer and remains the most important treatment modality for curative intent. Refinements in operative techniques along with the use of adjuvant radiotherapy and advanced chemotherapeutic agents have facilitated increasingly focused breast cancer operations. Surgical management of breast cancer has shifted from extensive and highly morbid procedures, to the modern concept obtaining the best possible cosmetic result in tandem with the appropriate oncological resection. An ever-growing comprehension of breast cancer biology has led to substantial advances in molecular diagnosis and targeted therapies. An emerging frontier involves the breast cancer microenvironment, as a thorough understanding, while currently lacking, represents a critical opportunity for diagnosis and treatment. Collectively, these improvements will continue to push all therapeutic interventions, including operative, toward the goal of becoming more focused, targeted, and less morbid.

## 1. Introduction

**Figure 1:**
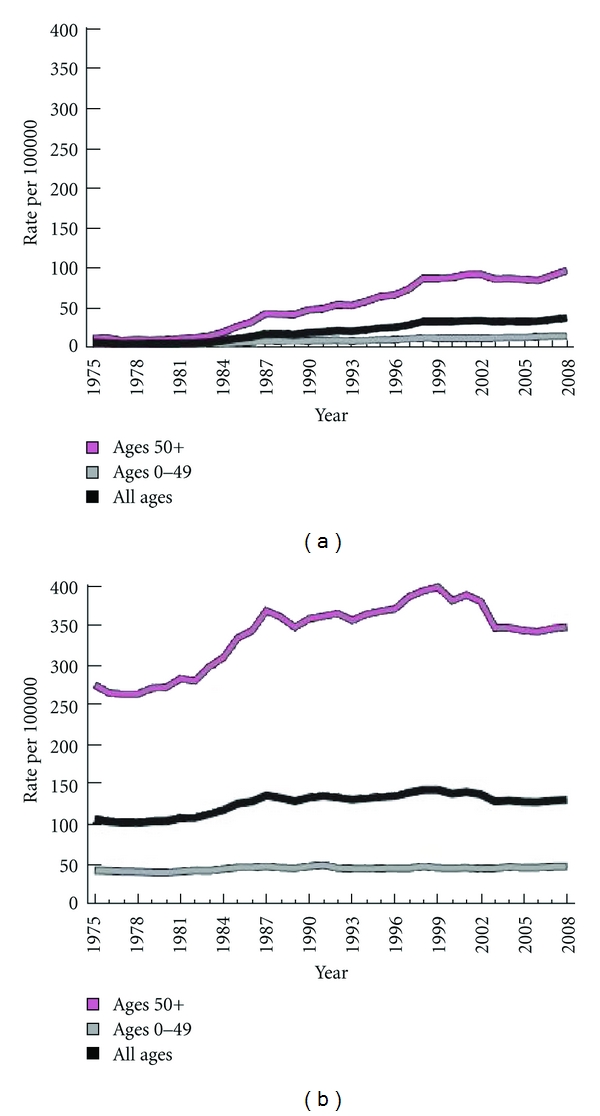
Incidence rates of In situ (a) and Invasive (b) female breast cancer in the United States (1975–2008). American Cancer Society. *Breast Cancer Facts and Figures 2011-2012*. Atlanta: American Cancer Society, Inc.

**Figure 2:**
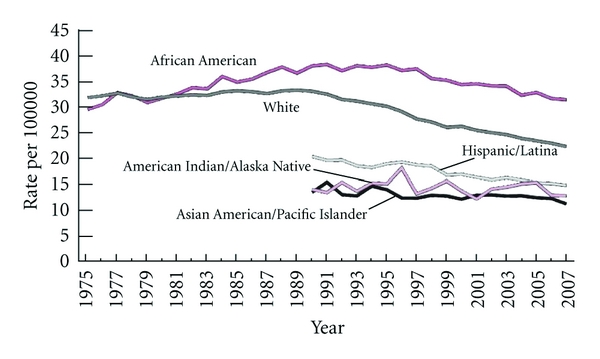
Mortality rate of female breast cancer, by race and ethnicity (1975–2007). American Cancer Society. *Breast Cancer Facts and Figures 2011-2012*. Atlanta: American Cancer Society, Inc.

Breast cancer is the most frequently diagnosed nondermatological malignancy in women and ranks second only to lung in cancer-related deaths [1]. While the incidence has increased over the past decade, (Figures 1(a) and 1(b)) the mortality rate of breast cancer has gradually declined [2, 3] (Figure 2). This improved survival may stem from earlier detection as well as improved therapies [2, 3]. 

Surgical resection was one of the first effective treatments for breast cancer and continues to play a critical role in the treatment of this disease. A multidisciplinary approach is now standard of care, involving a coordinated effort with the surgeon working in concert with the medical and radiation oncologist to achieve the best possible outcome for each individual. Improvements in both the quality and quantity of life for victims of breast cancer can be attributed to the advances made in each of these disciplines. As with all cancers, earlier stage disease is more readily manageable than after significant advancement. It is these early-stage cancers in which the most significant improvements in the operative management has occurred. Adoption of breast conservation surgery has allowed an increased focus on the cosmetic outcome, during a time that has also witnessed improved survival. This represents a clear victory for breast cancer patients, which needs to be extended to breast cancer of all stages.

## 2. Historical Progression of the Surgical Therapy of Breast Cancer

The Greek physician Galen is considered to be one of the earliest advocates of surgical treatment, recommending wide excision of breast tumors nearly 2000 years ago. Galen, like his predecessor Hippocrates, also recognized that breast cancer should be considered a systemic disease. Hippocrates proposed that cancers were the result of an imbalance of the four basic humours-blood, phlegm, and yellow and black bile. He attributed an excess of black bile for postmenopausal women having a greater incidence of breast cancer, as premenopausal women were relieved of this excess black bile with regular menstruation [4]. Although primitive, this concept can be extended to the current breast cancer treatment paradigm: systemic control of the disease at a molecular level, with local control by surgical intervention. While we now know that “black bile” does not result in breast cancer, the most effective breast cancer management embodies this concept of breast cancer as a systemic disease.

Early operations to treat breast cancer were primitive and brutal. These procedures consisted of amputation followed by cauterization, performed as rapidly as possible to minimize hemorrhage. Unfortunately, patients surviving the initial surgical procedure would all too frequently die of fulminant sepsis. In the late 19th to early 20th century, the advances of general anesthesia and antiseptic techniques facilitated more extensive procedures. Some of the most dramatic changes in surgical therapy for breast cancer were pioneered by William Steward Halstead [5]. His approach to the mastectomy helped change the surgical therapy of the breast from a simple amputation to a formal procedure. His technique, now termed the “radical mastectomy,” involved en bloc resection of the breast, the pectoralis muscle, and the axillary contents. This procedure was as effective at initial local tumor control as any early technique, with the significant advancement of a dramatically decreased rates of recurrence that plagued Halstead's predecessors [5].

During Halstead's era, prior to any understanding or capacity for early diagnosis, initial presentation of profoundly advanced tumors was the norm. Accepting Halstead's basic principles, surgeons attempted progressively extensive resections for cure of widely disseminated tumors. This evolved into dissection of the neck, abdomen, and even the mediastinum to remove diseased lymph nodes. Around the same period, early methods for surgical staging were developed, yielding a basic classification of patients with tumors in which radical mastectomy was potentially curative and those with disseminated cancer not appropriate for attempted resection. However, it would not be until the 1940s when evidence from preoperative staging brought the futility of what had become “superradical mastectomies” into question [5].

Initial deviation from the tenets of Halstead began in the late 1930s with an initial push for preservation of the nondiseased breast tissue during cancer resection. Shortly thereafter, postoperative radiotherapy was added for control of local tumor recurrence, laying the groundwork for breast conservation therapy (BCT) as we know it today [6]. Although BCT was not significantly implemented in clinical practice until the 1980s, the stage was set for the current surgical treatment of breast tumors utilizing either BCT or mastectomy.

## 3. Current Operative Management of Breast Cancer

Optimal management of a patient with breast cancer includes establishing a pathologic diagnosis prior to any definitive operative intervention. Formal surgical excision in the operating room is rarely required to establish the diagnosis of breast cancer, as there are many alternative techniques to obtain tissue for diagnosis. For example, much pathologic information can be gained from small, 1-2 mm “core” samples, allowing precise recommendations for treatment. The diagnosis of breast cancer is confirmed by histological evaluation, and the tumor is assessed for grade as well as human epidermal growth factor receptor 2 (HER2), estrogen, and progesterone receptor status [7]. This information is critical for optimal decision making regarding treatment options, most importantly allowing for coordination of care for those patients that will benefit from neoadjuvant chemotherapy prior to operative intervention [7].

After the diagnosis of breast cancer is established, patients are evaluated to determine the extent of the disease. Standard of care includes bilateral mammography to identify any suspicious areas in either breast that will impact surgical management. Laboratory values that will assist in treatment recommendations include complete blood count, liver function tests, and alkaline phosphatase. There are not established tumor markers for breast cancers, although cancer antigen (CA) 15-3 and CA 27-29 may be helpful when elevated. Additional imaging studies to evaluate for metastatic disease are obtained depending on signs and symptoms of the patient, as well as the clinical stage at presentation. A bone scan is indicated if the patient has localized bony pain or elevated alkaline phosphatase, chest imaging is indicated for pulmonary symptoms, and abdominal imaging by computerized tomography is indicated for abnormal liver functional tests or abdominal symptoms. A review of the acquired data, including pathology, laboratory assessment, and imaging, allows the multidisciplinary team to make recommendations for definitive management of the patient with breast cancer. Those patients with evidence of advanced disease are typically managed medically with preoperative chemotherapy, prior to any definitive surgical management.

Locoregional (operative) control of breast cancer remains the mainstay of treatment. Surgical treatment should allow the patient to be involved in the decision-making process, with the surgeon providing information about all surgical options available. Definitive surgical management typically involves breast conservation (BCT) or mastectomy. Local excision alone is at times acceptable, usually in the setting of elderly or otherwise debilitated patients without adjuvant radiation. This decision must be carefully weighed and based on evaluation of tumor aggressiveness and comorbid conditions of the patient.

There are two required components for BCT. First, tumors must be resectable with a pathologically clear margin, that is, a surrounding margin of breast parenchyma without disease. Secondly, patients undergoing partial mastectomy typically receive whole breast irradiation to achieve local control in the breast. Tumor size must be sufficiently small relative to the entire breast, such that the appearance of the breast is cosmetically acceptable following partial mastectomy. Additionally, all suspicious findings on imaging must be resectable with the partial mastectomy. The presence of diffuse highly concerning microcalcifications on mammography is a contraindication to BCT. Pregnancy and a history of previous chest irradiation do not allow BCT, as they are contraindications to the requisite postoperative radiotherapy. Positive margins after BCT require a repeat attempt at excision or completion mastectomy to achieve clear margins. Findings of involved margins with partial mastectomy significantly increase the chance of disease recurrence [8].

 Mastectomy is indicated for the curative resection of tumors (i.e., absence of metastatic disease) not amenable to BCT, and for those patients that do not want to consider conservation even though they meet criteria. The modern version of this procedure is termed the “modified radical mastectomy,” which entails removal of the breast, its underlying pectoralis fascia, and axillary contents, performed for more extensive disease.

In addition to resection of the primary tumor, all invasive breast cancers require assessment of axillary lymph nodes for tumor invasion. The ipsilateral axillary lymph nodes are theoretically the first site that breast cancer is expected to spread, with the sentinel nodes representing the first group of nodes at risk for invasion. Assessment of the axillary nodes includes sentinel lymph node biopsy (SLNB) during lumpectomy, or at the time of mastectomy. The SLNB represents another hallmark of targeted surgical therapy. Injection of a dye and/or radio-isotope into the breast allows the surgeon to identify the first (“sentinel”) lymph node draining the tumor basin. Involvement of axillary nodes is considered regional disease (not metastatic) and is usually followed by complete axillary node resection [8]. Nodal status provides critical staging information necessary for the proper selection of adjuvant therapy. Furthermore, negative findings after a properly performed SLNB allow a patient to avoid the potential for significant morbidity after axillary dissection. An all too common and often debilitating complication of this procedure is upper extremity lymphedema [9].

In situ breast cancer is a neoplasm that is completely contained within its basement membrane. This early neoplasm can be derived from a duct or lobule and is, therefore, referred to as lobule carcinoma in situ (LCIS) or ductal carcinoma in situ (DCIS). LCIS of the breast requires special consideration, as it is considered a marker for the future development of invasive breast cancer. The risk of developing invasive cancer is low, and if it occurs, histology tends to be favorable. For this group of women, LCIS is managed by appropriate monitoring without additional intervention. Alternatively, hormonal therapy can be administered for the purpose of breast cancer prevention. The potential adverse reactions of these medications must be considered and balanced with the presumed risk reduction.

In contrast to LCIS, the diagnosis of DCIS requires treatment for local control at the time of diagnosis. With the development of techniques for the earlier diagnosis of breast cancer, DCIS is the only diagnosis in approximately 15% of newly diagnosed breast cancer patients. This finding must be addressed, as the survival rates for treated DCIS are near 100%, but the development of invasive disease occurs in up to 30% of patients with untreated DCIS [10]. Treatment options include breast conservation with partial mastectomy and radiation, or total mastectomy. Although DCIS is often found in conjunction with an invasive carcinoma, treatment for the invasive component takes precedence and dictates both surgical and medical management. In contrast to management of invasive disease, those patients with DCIS usually do not require axillary dissection, as axillary nodal involvement in patients with pure DCIS is unusual. As a small number will have axillary involvement, sentinel node evaluation should be performed if mastectomy is the chosen operation for local control [11].

## 4. Breast Cancer Surgery and Chemotherapy

Starting in the mid-twentieth century, most notably in the lab of Bernard Fisher, early chemotherapeutic agents were being analyzed for use in the preoperative setting. The use of neoadjuvant chemotherapy (NACT) prior to an attempted surgical resection represents a dramatic improvement in breast cancer therapy, addressing the systemic aspect of this disease. NACT is indicated for locally advanced tumors or inflammatory breast cancer. Locally advanced breast cancer entails large tumors or those that invade the chest wall or skin (T4) or have spread to the axillary nodes (N2 or N3) [12].

An excellent response to chemotherapy merits reassessment of the patient to ensure a concomitant clinical and radiological response. Eradication of all tumor after neoadjuvant chemotherapy is termed pathological complete response (pCR), strictly defined as the absence of invasive cancer from the breast and axilla on pathological assessment in response to chemotherapy [13]. While achieving pCR has been found to increase long-term survival [14], a wide range of local recurrence rates (2.6–22.6%) after BCT following neo-adjuvant therapy has been noted [15]. One recent study indicates that Her2 positive and positive axillary lymphadenopathy may predict this recurrence after pCR [15]. While high risk populations certainly merit close postoperative surveillance for recurrent disease. Appropriately placing those patients achieving excellent response to chemotherapy into the algorithm for the surgical management of breast cancer requires further assessment. Improved methods are needed to predict those tumors best amenable to downstaging to BCT, as certain patients may in fact be better candidates for mastectomy. Furthermore, strict criterion defining the medical management of successful pCR is also needed. Molecular tests such as the 21 gene (oncotype DX) and 70 gene (mammaPrint) assay [7], that provide tumor-specific scores reflecting risk of recurrence, may become useful in this scenario.

The effectiveness of NACT for locally advanced disease eventually led to the use of pre-operative treatment in an attempt to “downstage” even more advanced cancer to a scope amenable to treatment by mastectomy [12]. A recent extension of these principles is the use of chemotherapy to downstage tumors, in order to avoid mastectomy altogether in lieu of BCT. NACT is indicated for tumors meeting all criteria for breast conservation (see above) except for tumor size. An excellent response in this scenario has now allowed the option for BCT in a patient who would have required a mastectomy.

## 5. Recent Advances in the Surgical Therapy of Breast Cancer

Most of the recent advances in the surgical management of breast cancer follow the basic template of ever more conservative surgical resections. The first involves operative breast cancer therapy with a concomitant focus on breast reconstruction, known as oncoplastic breast surgery [16]. This trend represents another advancement made possible by the refinements in the use of postoperative radiation, the same concept that led to the advent of BCT. Oncoplastic surgery entails the use of plastic surgery techniques to restore cosmesis and natural symmetry, ideally during cancer resection [16]. Plastic surgery techniques utilized include breast augmentation and reduction, flaps, implants, and expanders, on both the diseased and the normal breast if necessary to achieve the desired symmetry. Indications are still widely debated, but appropriate candidates are those that have sufficient residual breast after the oncological resection to facilitate the necessary reconstruction [6].

One of the most recent advances in surgical therapy involves management of the positive sentinel lymph node biopsy (SLNB). Traditionally, a positive SLNB represents an absolute mandate for a complete axillary dissection. Substantial morbidity, not unlike that which was seen in the days of Halstead, all too often follows. However, a recent study has demonstrated that high-risk patients with small tumors (T1-T2) and limited lymph node spread, who are able to receive radiotherapy, do not benefit further from complete axillary lymph node removal [17]. Simply stated, survival for small breast tumors with limited spread does not improve after axillary dissection in older individuals or those with significant medical problems. This early work has found that this subset of patients suffer more from the complications of the procedure than benefit. The adjuvant radiotherapy therapy given for this early-invasive disease seems to provide most of the survival benefit.

A recent trend including surgery as cancer prevention has gained wide acceptance. Contralateral prophylactic mastectomy (CPM) has been found to decrease the risk of development of a cancer in the disease-free breast in women at high risk. Those women harboring a BRCA mutation or a strong family history of breast cancer may be considered candidates for prophylactic bilateral mastectomy. As mentioned previously, with the diagnosis of LCIS, the risk of developing an invasive breast cancer is equal in both breasts, such that bilateral mastectomy may be necessary for true risk reduction. Many women, in an otherwise low-risk category, also opt for CPM after a newly diagnosed breast cancer. This usually involves fear of developing disease in the contralateral breast. While recent data suggests an increased overall as well as disease-free survival after CPM [18], the debate is ongoing regarding the appropriate indications for CPM. This is in fact an extensive operation with the potential for significant morbidity. The decision to take such a measure is formidable. Similarly, the quest to identify the population benefiting the most from this intervention must be equally rigorous.

## 6. Surgery and Breast Cancer Metastasis

The most successful operative management of metastasis is prophylactic: appropriate screening for detection of suspicious lesions of the breast, followed by appropriate local control to minimize the potential for metastatic dissemination. This is reflected in recent trends showing improved survival of breast cancer patients, as screening and early intervention has translated into improved outcomes. After the diagnosis and completion of treatment of a primary breast cancer, surveillance for recurrence or metastatic spread ensues. Followup entails focused clinical and laboratory assessment, and mammography to detect new or recurrent lesions.

The discovery of metastatic disease at any point merits a complete reevaluation. Traditionally, surgical intervention was avoided in the patient harboring metastatic disease, due to a perceived lack of benefit. Only those patients with extremely limited metastatic lesions were considered for therapeutic resection. For the most part, patients found to have metastatic disease were deferred to induction chemotherapy in the hopes of an excellent response and prolongation of life.

Most traditional use of surgical intervention in the setting of metastatic disease was for palliative purposes, at either the primary tumor site or any distant metastatic lesions. For example, resection of the primary tumor was considered for persistent infection, bleeding, or general difficulty maintaining cleanliness. However, many recent studies have been able to challenge this practice of avoiding intervention on the primary tumor in the setting of metastatic disease. Early studies indicate that resection of a primary breast lesion may increase survival in the setting of limited metastasis. This effect probably stems from more effective and specific chemotherapy, but randomized trials are needed to define both the optimal candidates and indications for this intervention. However, the significance of the early findings of reduced need for chemotherapy, improved quality of life, and even long-term cures with the concomitant resection of (limited) metastatic lesions cannot be overstated [19].

Operative intervention for metastasic lesions is typically palliative, involving the treatment of a symptomatic mass. This may entail bypassing an obstructing metastatic lesion in the bowels, utilizing a normal segment of bowel to allow free flow of intestinal contents. However, aggressive resection of metastatic lesions for curative intent has gained favor in recent years. The best studied is the resection of metastatic lesions to the lung, in which long-term success and even some cures have been reported. The patients most amenable to metastasectomy are those with limited metastatic burden (oligometastases) with hormonally responsive tumors. Operative characteristics include smaller lesions in a location that facilitates complete removal [20].

It is a well-known fact that the most common site of breast cancer metastasis is the bone, with breast cancer being the leading cause of bone metastasis of any cancer in women. The lung, liver, and brain are other common sites of metastasis. However, it has recently been demonstrated that the basic breast cancer subtypes (Luminal A, luminal B, HER-2 positive, and basal) differentially target certain sites for metastasis. For example, the HER-2 positive and triple negative subtypes have been shown to preferentially metastasize to the brain over the other subtypes [21]. While this represents an interesting finding, further investigation is needed to translate this data into clinical practice. For example, knowledge of the presence of a basal phenotype in a high risk patient may merit more aggressive, organ-specific followup.

## 7. Surgery and the Breast Cancer Microenvironment

Surgical resection of breast cancer is absolutely curative if performed while the primary tumor is contained. Escape of tumor cells from the primary lesion completely changes therapeutic management, expectations, as well as outcomes. Chemotherapy becomes the primary hope for cure as opposed to surgical intervention. Interestingly, some early stage tumors, all of which were previously assumed to be self-contained, have been shown to harbor the capacity for systemic tumor dissemination. While there is no method to accurately predict which tumors have this devastating capacity, certain factors such as large tumors, younger age at diagnosis, vascular invasion, and nodal involvement have been found to be associated with a high risk of developing distal metastasis after appropriate treatment [22, 23].

The best treatment option currently available is effective loco-regional control of the primary tumor. The surgeon's primary focus at the time of resection is obtaining clear margins. Most studies have found that obtaining at least a 2 millimeter margin for invasive and in situ breast cancer best minimizes the chance of local recurrence [24, 25]. This threshold has consistently led to reduced local recurrence rates, while balancing the potential for an overly aggressive resection. Effective local control removes the nidus for both local and distant recurrence, emphasizing the management of the primary tumor on the systemic aspect of the disease. This effect is exemplified by the significant increase in distant metastasis rates and subsequent survival with the development of a local recurrence of a resected breast tumor [22–26].

Further evidence that breast cancer, even at its early stages, can be a systemic disease can be found in animal studies and early analysis in cancer patients. Utilizing PCR and immunohistochemistry, increased cancer-related cells have been demonstrated in the systemic circulation due to surgical manipulation [27–30]. Needle biopsies of primary tumors have even been found to result in increased rates of nodal metastasis [31, 32]. Tumor cells that break off from the primary site and enter the systemic circulation are referred to as circulating tumor cells (CTCs). While the CTCs were first described over a century ago, the technology for their detection has only recently become reliable. Current methods allow for the enrichment of CTCs by antibody-mediated targeting of the epithelial cell adhesion marker (EpCAM). While the clinical usefulness of CTC assessment is controversial, some consider that greater than five CTCs is the breaking point for a poor prognosis in breast cancer [33, 34].

Detection of CTC has been used to demonstrate significant shedding of putative tumor cells into the systemic circulation during surgical manipulation [35]. While this shedding is known to occur in both breast and lung cancers [36], the functional result and ability of these cells to successfully migrate and seed distant sites is not known [37]. Furthermore, some hypothesize that the tissue trauma resultant from needle or operative manipulation may lead to the expression of an invasive or metastatic phenotype [38]. This alteration may lead to cancer progression or the release of CTCs, respectively [39]. Pathways implicated in these effects are normal and appropriate wound healing responses, such as those involved in inflammation and angiogenesis [38]. With the continued technological improvement for the detection of CTCs, determining the clinical relevance of these effects may become possible. The assessment of CTCs could one day provide the basis for highly specific real-time biopsies, yielding a strong potential for the modification of surgical techniques and traditional indications. The capacity to harvest and analyze CTCs could become a key feature of individual tumor profiling, allowing for patient-specific therapies to further reduce the current complication profile of today's interventions [8, 40].

## 8. Conclusion

Surgical intervention is currently the best hope for definitive cure of breast cancer. Even so, recent advances represent significant steps away from the extensive resections performed by Halstead and his predecessors. While these early attempts successfully decreased local recurrence rates, advances in the treatment of breast cancer as a systemic disease were needed to facilitate long-term cures. Continued improvements in early diagnosis via breast imaging, advanced prognostic tests, patient-specific molecular diagnosis, and the development of targeted chemotherapeutic agents provide hope for improved survival rates. By doing so, breast cancer therapy will become more focused, increasing efficacy and reducing complications of all the treatment disciplines. This will move the bar closer to the ultimate goal of transforming breast cancer into an easily targeted, readily manageable disease.
